# Supplementary material for: The Association between Caffeine Intake and the Colonic Mucosa-Associated Gut Microbiota in Humans—A Preliminary Investigation
Source: Nutrients. 2023 Apr 3;15(7):1747. doi: 10.3390/nu15071747 (PMC10096519; doi:10.3390/nu15071747)
Supplement: Supplementary file 1 [file nutrients-15-01747-s001.zip › nutrients-2284706-supplementary.pdf]

Supplemental Table S1. Basic characteristics of study participants based on coffee consumption.

**Characteristics Mean ± standard deviation or n (%)**	**< 2 cups (n=17)**	**2 cups (n=9)**	**≥ 3 cups (n=8)**	***P* value**
Caffeine (mg)	44.7 ± 33.6	106.3 ± 41.0	163.8 ± 71.4	< 0.0001
Age (years)	61.4 ± 5.16	64.2 ± 4.38	60.7 ± 7.35	0.38
Men, n (%)	17 (100%)	9 (100%)	7 (87.5%)	0.24
Racial Group				0.18
Non-Hispanic white, n (%)	12 (70.6%)	9 (100%)	7 (87.5%)	
Body mass index (kg/m^2)	33.6 ± 6.32	36.4 ± 7.23	31.9 (5.54)	0.34
Smoking Status, n (%)				0.83
Never smokers	8 (47.1%)	2 (22.2%)	3 (37.5%)	
Former smokers	6 (35.3%)	5 (55.6%)	3 (37.5%)	
Current smokers	3 (17.6%)	2 (22.2%)	2 (25.0%)	
Alcohol Status, n (%)				0.86
Never drinkers	4 (23.5%)	2 (22.2%)	3 (37.5%)	
Former drinkers	6 (35.3%)	3 (33.3%)	1 (12.5%)	
Current Drinker	7 (41.2%)	4 (44.4%)	4 (50.0%)	
Hypertension, yes, n (%)	12 (70.6%)	8 (88.9%)	5 (62.5%)	0.47
Diabetes, yes, n (%)	8 (47.1%)	5 (55.6%)	4 (50.0%)	1.00
Daily total calorie intake (kcal)	1839 (±605)	1632 (±644)	2301 (±853)	0.13
Total carbohydrate (gram/1000kcal/day)	115 (±23.2)	112 (±18.3)	112 (±19.0)	0.89
Total protein (gram/1000kcal/day)	36.1 (±5.99)	40.5 (±8.43)	40.0 (±8.21)	0.25
Total fat (gram/1000kcal/day)	41.5 (±8.29)	42.5 (±6.86)	43.4 (±5.07)	0.82
Vitamin B2 (mg/1000kcal/day)	0.89 (±0.21)	1.20 (±0.22)	1.34 (±0.37)	0.0005
Vitamin B6 (mg/1000kcal/day)	1.48 (±0.50)	1.66 (± 0.71)	2.22 (± 0.90)	0.07
Vitamin B12 (mcg/1000kcal/day)	2.19 ((±0.62)	2.72 (± 1.07)	2.82 (±0.93)	0.15
HEI score[1]	59.8 (±9.22)	61.8 (±9.65)	62.4 (±8.41)	0.76

[1]HEI, healthy eating index.

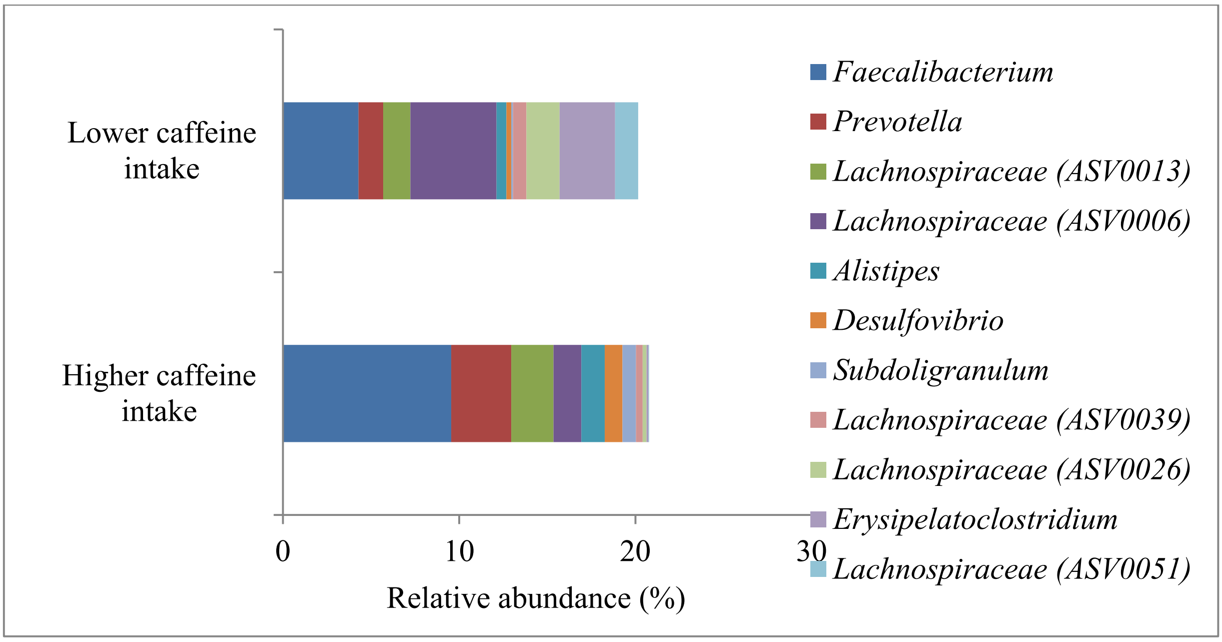

Supplemental Figure S1. The relative abundance of bacterial genus by lower vs. higher intake of caffeine (q value < 0.05).
